# Xenophobia amidst and post‐COVID‐19 pandemic

**DOI:** 10.1002/hsr2.1252

**Published:** 2023-05-04

**Authors:** Alishba Adnan, Fatima B. Athar, Abubakar Nazir, Arpit Mago, Sidhant Ochani, Amna Siddiqui

**Affiliations:** ^1^ Department of Medicine Karachi Medical and Dental College Karachi Pakistan; ^2^ Department of Medicine King Edward Medical University Lahore Pakistan; ^3^ Department of Medicine Jawaharlal Nehru Medical College Belgaum Karnataka India; ^4^ Department of Medicine Khairpur Medical College Khairpur Mir's Pakistan

Xenophobia refers to the fear of anything viewed as being odd, fear of the strange, or unknown such as an intense dislike of practices, and people having an outsider status.^1^ They express mistrust, avoid interacting with and blame perceived outsiders for local issues, treat them differently such as by being hostile to them, and treat them as dangerous without having any proof.^2^ The Diagnostic and Statistical Manual of Mental Disorders does not list xenophobia as a mental illness (DSM‐5). This behavior was common among children/adolescents amidst and post‐COVID‐19 pandemic.

The COVID‐19 pandemic afflicted the world and resulted in grave psychosocial issues in children, and the mental state of many has been negatively impacted by current issues. Managing the outbreak with quarantine measures such as lockdowns led to children having limited social interactions. According to a study, children who remain inhibited between the ages of 21 months and 7.5 years, had a higher risk of developing anxiety disorders compared to children who were uninhibited or unstably inhibited.^3^ With mandatory lockdowns, children born or brought up during the pandemic had “unstable inhibition” and never had a constant socializing experience. Countries have correctly noted the connection between COVID‐19 and psychiatry in their work, especially children who experienced unpleasant emotions, mood swings, and alterations in their sleeping and feeding schedules were more likely to relapse into mental illness as well as exacerbate pre‐existing mental health conditions.^4^

People have been subjected to discrimination and xenophobic views and have suffered from social and economic inequality.^5^ Migrants in search of a better quality of life are considered a source of pathogens causing disease outbreaks which have exposed them to heightened discrimination and stigmatization.^6^ This inflicts a negative impact on the mental health of children too when parents are exposed to discriminatory attitudes. The discrimination against Asians and Africans was more profound with verbal and physical attacks against them on the rise during the COVID pandemic reflecting the worsening of racism‐related stigma.^7^, ^8^ According to reports received by United States‐based Stop AAPI Hate coalition, there were 10,370 cases of hate crimes against Asian American and Pacific Islanders (AAPI) during the pandemic (2020–2021).^9^

There was a horrifying 50% rise in hate crimes in 2 years as announced by the United Kingdom‐based organization End Violence and Racism against the East and Southeast Asian Communities (EVR). This segues devastating consequences such as suicide attempts, depression, and social isolation, especially among the vulnerable adolescent population. This was further provoked by xenophobic bullying (in person and over the internet) in schools with terms like “Chinese flu,” which affected the victims’ mental health devastatingly.^10^

The impact of COVID‐19 on children was worsened due to disruption to education mostly by the closing down of schools, which impacted the daily routine of children and was a halt in their developing social skills. The pattern of sleeping without waking up early gave rise to monotony, distress, impatience, annoyance, and varied neuropsychiatric manifestations. So even when the schools did open, children had trouble adjusting to their new routines and hence fell prey to social anxiety and tended to avoid interacting with peers. Lack of supervision, emotional issues from unstructured schedules, and diminished peer connection cause a detrimental effect on the long‐term development of Children born to immigrant families (CIF).^11^

The rising levels of unemployment also impact children directly, putting their mental health and safety at risk, and predisposing them to likely domestic abuse. It is to be noted that mothers, under the pressure of financial constraints, also fall into depression and anxiety. According to a study,^12^ maternal social phobia expects an increasing fear of strangers among children of 10–14 months of age,^13^ and maternal depression predicts even greater increases in fearfulness between 4 and 12 months of age.^14^ Incidences of domestic violence, child abuse, and adulterated online content are on the rise. Besides, children from marginalized and migrant communities are soft targets of xenophobia and suffer from hazardous consequences, including child labor, child trafficking, child marriage, sexual exploitation, death, and so on.^15^

Discrimination against migrant communities creates unhealthy living environments both physically and as well mentally impacting children the most, making them afraid of the unhealthy social environment that they are forced to live in. The causes of xenophobia implicate their very survival as healthy adults very difficult. Adolescents delay seeking the psychological help that they need for mental health disturbances during the pandemic, and as a result, attempted and successful suicide rates were higher during the pandemic period. There were a total of 5568 deaths by suicide centered around the youth population in America in 2020. This population was 79.2% male, 59.6% non‐Hispanic White and the most common cause of death was by use of firearms (51.1%).^16^

It is suggested that educational arbitrations should be done through trusted health workers, celebrities, clergy, and journalists to decrease stigma among school‐going children. The spread of appropriate information should be done via professional groups to mitigate the burden of xenophobia. Psychoeducational therapy, workshops, and support teams in schools are beneficial means for mental health advocacy and mitigating the effects of xenophobia among children. There should be antibullying campaigns and a zero‐tolerance policy practiced strictly at schools. Any form of cyberbullying should be encouraged to report to cyber security or at least parents. Parents should monitor their child's access to and exposure to social media. Some news channels and digital platforms can aggravate xenophobia in children by spreading fabricated and biased information. Furthermore, children should be encouraged to look for explicit information from well‐grounded sources.

Education regarding the transmission of disease and the spread of public health information to guardians and children can decrease xenophobia. Parents should model positive behavior, children are keen learners and observers, so parents should demonstrate respect and kindness in their language and deeds. Right information should be conveyed by the media for promoting solidarity, unity, and hope and decrease discrimination.

Nonnational students in schools and children who are stranded in other countries due to the closure of borders should be given equal rights to state services like education without any discrimination. Communication strategies like interactive sessions among children of all races and volunteer activities should be enhanced to encourage social unity during the COVID‐19 pandemic. These activities aim to minimize the gaps among children from diverse backgrounds. This can be achieved by ensuring equality in information access in schools, equal opportunities for children in education schools, and minimizing inequalities in media coverage concerning race and nationality. Moreover, children should be exposed to different people belonging to diverse origins. It can be done by attending various cultural events, playdates, and festivals to help children appreciate and acclimatize to diversity.

Children should be encouraged to participate in volunteering and welfare services that will enable the children to interact with people from different regions and help to figure out their needs. It can be done by volunteering in community centers, food banks, and animal shelters. The most important of all is to address the concerns and fears of children suffering from xenophobia. They should be helped by the parents and teaching staff in schools in developing various coping strategies and also to be more expressive about their concerns. It is also recommended to seek help from mental health professionals for children experiencing mental health issues like xenophobia to improve their emotional well‐being.

## AUTHOR CONTRIBUTIONS

**Alishba Adnan**: Conceptualization; methodology; project administration; resources; writing—original draft. **Fatima B. Athar**: Investigation; writing—original draft. **Abubakar Nazir**: Validation; writing—original draft. **Arpit Mago**: Methodology; writing—original draft. **Sidhant Ochani**: Data curation; supervision; writing—original draft; writing—review and editing. **Amna Siddiqui**: Data curation; validation; writing—original draft; writing—review and editing.

## CONFLICT OF INTEREST STATEMENT

The authors declare no conflict of interest.

## ETHICS STATEMENT

No animals or human subjects were used in the current study, only publicly available data was used, and no ethical approval is needed. All authors have read the final manuscript and given consent for publication.

## TRANSPARENCY DECLARATION

The lead author (Sidhant Ochani) affirms that this manuscript is an honest, accurate, and transparent account of the study being reported; that no important aspects of the study have been omitted; and that any discrepancies from the study as planned (and, if relevant, registered) have been explained.

## Data Availability

Data and materials can be retrieved from the corresponding author as per reasonable request.
